# Long-root *Eichhornia crassipes* waste plants dual-purpose resource utilization: green preparation of magnetic carbon quantum dots for heavy metal deep removal

**DOI:** 10.1186/s40643-026-01064-x

**Published:** 2026-04-28

**Authors:** Yihong Guo, Mingxin Cui, Hongjun Yang, Jun Chen, Sen Lin

**Affiliations:** 1 Salt Lake Chemical Engineering Research Complex, Qinghai University, Xining, China; 2 National Engineering Research Center for Integrated Utilization of Salt Lake Resources, East China University of Science and Technology, Shanghai, China; 3 Yunnan Research Institute of Ecological Agriculture, Kunming, China

**Keywords:** Adsorption, Carbon quantum dots, Heavy metals, Long-root *Eichhornia crassipes*, Magnetic nanopartion

## Abstract

Long-root *Eichhornia crassipes*, an aquatic plant with extensive root systems, has demonstrated potential in remediating eutrophicated water bodies. However, improper disposal of large quantities of discarded plants post-remediation can easily cause secondary pollution. In this study, rapidly recoverable magnetic carbon quantum dots (MCQDs) were greenly synthesized via hydrothermal treatment of long-root *Eichhornia crassipes* and subsequent coupling with Fe_3_O_4_ nanoparticles. Adsorption experiments demonstrated that MCQDs displayed a commendable adsorption capacity for various heavy metals in municipal wastewater under the synergistic effect of CODs and Fe_3_O_4_. Mechanistic analyses revealed that the adsorption involved chemical binding with oxygen-containing functional groups and amino groups on the surface of MCOQs. The saturated MCQDs were efficiently recovered within 5 min using an applied magnetic field, offering a practical solution for nanoparticle recovery. This study provides valuable insights into the green resource utilization of Long-root *Eichhornia crassipes* based on CQD technology.

**Graphical abstract:**

**Supplementary Information:**

The online version contains supplementary material available at 10.1186/s40643-026-01064-x.

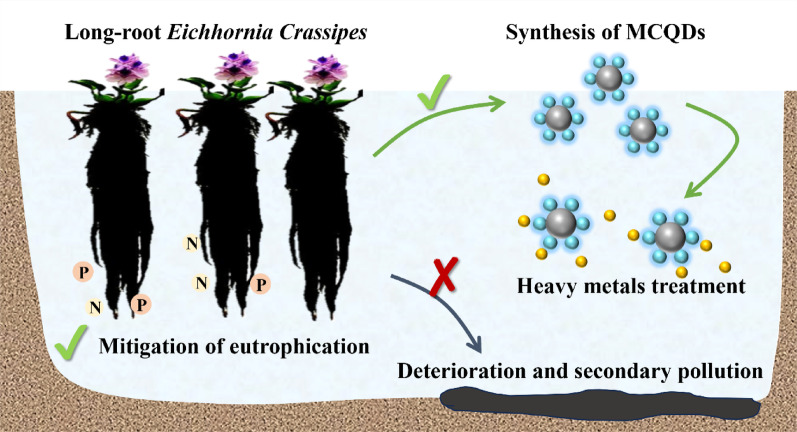

## Introduction

Long-root *Eichhornia crassipes* is a recently developed variety of water hyacinth employed to mitigate eutrophication of aquatic ecosystems (Lin et al. 2018; Liu et al. 2021). Compared with traditional plants, it has a more developed root system, accounting for more than 80%, and an excellent ability to remove nitrogen and phosphorus to limit the malicious spread of algae in the water (Liu et al. 2018). However, the harvested plants after wastewater treatment often accumulate in large quantities, leading to secondary pollution and disposal challenges if not properly managed. Consequently, how to deal with waste plant and recycle it as a usable resource is a pressing challenge (Lin et al. 2012, 2022). Due to its well-developed root system and low moisture content, current research focuses on its application in the preparation of activated carbon or other modified adsorbents (Cao et al. 2019; Lin et al. 2020). Converting waste biomass into functional nanomaterials offers a promising solution that addresses both waste disposal and pollution remediation.

Carbon quantum dots (CQDs), less than 10 nm in diameter, have abundant functional groups on surface, good fluorescence characteristics, biocompatibility, and non-toxicity, enabling their wide application in environmental, biomedical, bioenergy, and food safety fields (Wang and Hu 2014; Chen et al. 2021a, b, c; Luo et al. 2021). Preparation methods of CQDs can be divided into the “Top-down” method that converts bulk carbon materials into nanoparticles by oxidation, reduction, stripping and shearing, and “Bottom-up” approaches that fuse or carbonize small molecular compounds by high-temperature pyrolysis and spontaneous chemical reactions (Tian et al. 2009; Yang et al. 2009; Bao et al. 2011). However, existing methods for CQDs synthesis often involve difficult reaction conditions, high costs, cumbersome processes, and toxic raw materials, limiting their scalable preparation and practical application.

The usage of green and natural substances as synthetic raw materials may overcome these disadvantages and be additionally valuable (Mahesh et al. 2016; Jia et al. 2024). As a green and biocompatible nanomaterial, carbon quantum dots derived from diverse carbohydrate sources have shown great potential for broad applications across environmental, biomedical, and food safety fields (Berdimurodov et al. 2025). Selecting the roots of waste Long-root *Eichhornia crassipes* plants as raw material not only enables environmentally friendly CQDs production but also offers a sustainable solution for repurposing waste biomass reuse and heavy metals pollution treatment.

In this work, CQDs generated by waste Long-root *Eichhornia crassipes* plants can be uniformly doped with superparamagnetic nanoparticles (Fe_3_O_4_ or γ-Fe_2_O_3_) to create hybrid magnetic CQDs (MCQDs), which can be used for heavy metal removal in the water bodies. The physicochemical properties of MCQDs were comprehensively characterized, and their magnetic recovery behavior was investigated through kinetics measurement. Moreover, the adsorption performance was evaluated through kinetics and thermodynamic experiments, based on which the mechanism of efficient heavy metal adsorption by MCQDs was determined. This work demonstrated a sustainable strategy wherein waste Long-root *Eichhornia crassipes* biomass, originally employed for ammonia nitrogen removal, is repurposed as a precursor for magnetic carbon quantum dots for heavy metal remediation, achieving dual-purpose benefits in both ammonia nitrogen pollution treatment and heavy metal remediation.

## Experimental sections

### Materials and reagents

All metal salt chemicals were purchased from Macklin Biochemical Technology Co., Ltd. Ammonium nitrate and sodium hydroxide were bought from Sinopharm Chemical Reagent Co., Ltd. All chemicals used in this study were of analytical reagent grade.

### Synthesis of Fe_3_O_4_

The synthesis was conducted in a jacketed reactor with a water bath temperature of 45 °C and a stirring rate of 300 rpm. 2.7062 g FeCl_3_ 6H_2_O and 0.9954 g FeCl_2_ 4H_2_O were dissolved in 100 mL of deionized water under stirring. After complete dissolution, 6 mL of NaOH solution (8 mol/L) was rapidly added. The mixture was stirred for 30 min, yielding a Fe_3_O_4_ suspension.

### Synthesis of MCQDs

Based on literature review and preliminary experiments on biomass-derived CQDs (Nguyen et al. 2022; Durairaj et al. 2022; Kunnath Parambil et al. 2025), CQDs were successfully synthesized via hydrothermal reaction of long-root *Eichhornia crassipes* root powder in deionized water at 180 °C for 10 h. The products were centrifuged (10000 rpm, 20 min) to obtain the CQDs liquid. Subsequently, the CQDs solution was thoroughly combined with a Fe_3_O_4_ suspension prepared by co-precipitation of a Fe (III)/Fe (II) mixture in a NaOH solution to obtain MCQDs.

### Adsorption kinetics

All adsorption kinetics experiments in this work were conducted at 30 °C, with the mass ratio of MCQDs adsorbent to heavy metal solution meticulously maintained at 0.5 g per 150 mL. Samples were collected at appropriate intervals and analyzed to determine the concentration of heavy metal ions. The adsorption capacity *q*_e_ (mmol/g) was calculated according to Eq. (1)

1 $$ q_{{\mathrm{e}}} = \frac{{\left( {C_{0} - C_{{\mathrm{t}}} } \right)V}}{m}~ $$

where *C*_0_ and *C*_t_ were the beginning and residual concentrations (mmol/L) of heavy metal in solution, respectively, *V* was the adsorption solution volume (L), and *m* was the mass of MCQDs (g).

### Adsorption isotherm

Adsorption isotherm measurements were carried out at 15, 30, and 50 °C using heavy metal solutions with initial concentrations ranging from 0 to 1 mmol/L. The duration of experiments was set at 10 h to ensure that the adsorption process reached equilibrium.

### Magnetic recovery

For the magnetic recovery experiments, three samples were prepared by dispersing 0.1, 0.2, and 0.3 g of MCQDs particles in 100 mL of solution, respectively. Each suspension was placed under a static magnetic field generated by a 0.1 T permanent magnet for 5 min. The magnetic recovery was determined by measuring the iron element of unrecovered MCQDs in the suspension. The magnetic recovery ratio *η* was calculated according to Eq. (2).

2 $$ \eta = \left( {1 - \frac{{c_{{\mathrm{t}}} }}{{c_{0} }}} \right) \times 100\% $$

where *c*_t_ was the Fe^3+^ concentration of the sample with unrecovered suspended MCQDs particles that were collected and dissolved at specified times, and *c*_0_ was the initial Fe^3+^ concentration of the uniform suspension.

### Assays

The photoluminescence (PL) spectra were recorded using a fluorescence spectrophotometer (LS-55, PerkinElmer, America). The surface situation of MCQDs was observed utilizing a scanning electron microscope (SEM, GeminiSEM 360, ZEISS, Germany). To further analysis of MCQDs, TEM images were acquired by the transmission electron microscope (Talos F200x, Thermo Fisher, America). Particle size analysis of MCQDs was performed using a laser particle size analyzer (Mastersizer 3000, Malvern, UK). Deionized water was used as the dispersant, and the obscuration level was controlled within 10–15% during the measurement. X-ray photoelectron spectra (XPS) of surface elements were obtained through the X-ray analyzer (Nexsa, Thermo Fisher, America) with greater than 3 μm of resolution. The pattern of MCQDs was obtained by X-ray diffractometer (XRD, SmartLab SE, Rigaku, Japan) with a scanning range of 5°-80°. Fourier transform infrared spectra were determined with an FT-IR spectrometer (Nicolet iS20, Thermo Fisher, America). The magnetic performance was tested on a vibrating sample magnetometer (7404, LakeShore, America). The thermal gravimetric analyzer (TGA, SDTQ600, TA, America) was conducted to measure the weight loss of MCQDs and Fe_3_O_4_ within the temperature range of 10–600 °C. The surface area and pore size distributions results of MCQDs were obtained by BET instrument (ASAP 2460, Micromertics, America). The metal ions concentrations were determined by an ICP-OES instrument (ARCOS, Spectro, Germany).

## Results and discussion

### MCQDs characterization

**Fig. 1:**
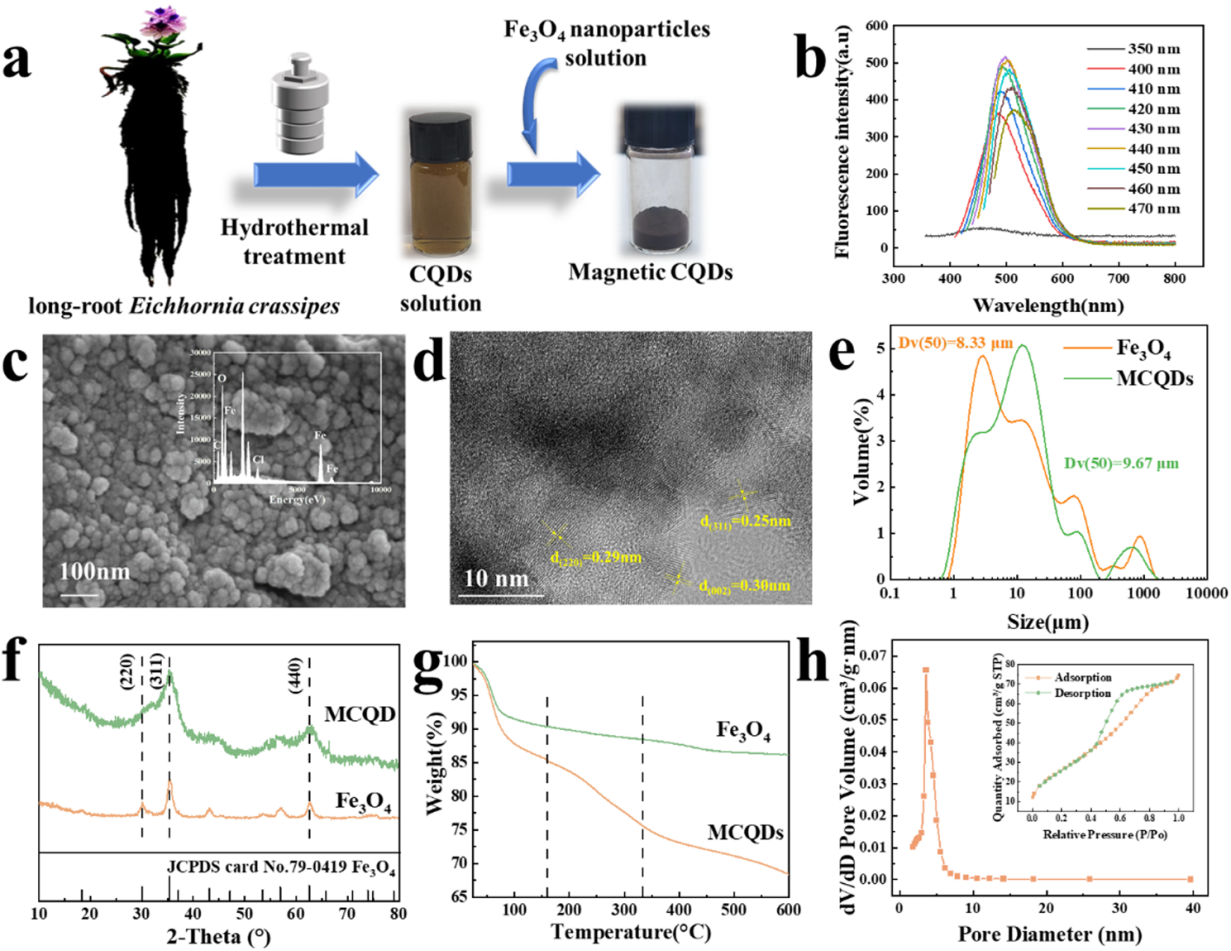
Synthetic schematic of MCQDs (**a**). The PL spectra of CQDs liquid (**b**). The surface morphology (**c**), HRTEM image (**d**), particle size distribution (**e**), XRD patterns (**f**), TG results (**g**), and N_2_ adsorption/desorption isotherms (measured at 77 K) (**h**) of MCQDs

As shown in Fig. 1a, a CQDs solution was prepared using long-root *Eichhornia crassipes* root powder and combined with magnetic substances to form magnetic CQDs. It could be seen in Fig. 1b that the CQDs solution exhibited photoluminescence behavior, with maximum emission at 500 nm under 430 nm excitation, confirming the successful preparation of the CQDs solution (Zhu et al. 2013). SEM images were shown in Fig. 1c. The MCQDs nanoparticles had a granular structure, but the particles were mostly clustered together with a wide range of particle size distributions due to the presence of Fe_3_O_4_ magnetism. Figure 1d showed the HRTEM images of MCQDs. The spacing of 0.25 and 0.29 nm, respectively, was found to be consistent with Fe_3_O_4_ (Chen et al. 2021a, b, c). The graphitic carbon with a lattice spacing of about 0.30 nm was at flanks of Fe_3_O_4_ particles (Ðorđević 2022). The elemental mappings were shown in Fig. S1, and the distribution of the elements was uniform and dense. The above results certified the good distribution of CQDs on Fe_3_O_4_.

Laser particle size analysis showed that bare Fe_3_O_4_ had a median particle size of 8.33 μm, while MCQDs had a median particle size of 9.67 μm, indicating that the co-precipitation process did not significantly alter the Fe_3_O_4_ particle size (Fig. 1e). It is well documented that Fe_3_O_4_ nanoparticles prepared by co-precipitation tend to form aggregates in solution while their primary particles remain in the nanoscale range (Abboud et al. 2015). As shown in Fig. 1f, the obvious characteristic peaks were consistent with Fe_3_O_4_ (JCPDS card No. 79-0419), confirming that the synthesis of Fe_3_O_4_ could be achieved through this methodology. However, due to the low CQDs content in the composite adsorbent, the characteristic peak of graphite was absent in the MCQDs. XRD analysis showed that the diffraction peaks of Fe_3_O_4_ in MCQDs became slightly broader compared to bare Fe_3_O_4_, which may indicate reduced crystallinity due to the interaction with CQDs. These results were consistent with our previous studies on magnetic materials, where functionalization of Fe_3_O_4_ typically does not alter their core structure (Lin et al. 2018; Chen et al. 2020, 2021a, b, c). The XRD results agreed with the emergency of the characteristic peaks in XPS spectra (Fig. S2a), which verified the well combination of Fe_3_O_4_ and CQDs in the MCQDs. The FT-IR spectra of the Fe_3_O_4_ and MCQDs were shown in Fig. S2b. After linking with CQDs, a new peak was observed at 1097 cm^−1^, which belonged to the alkoxy peak and at 2920 cm^−1^ showed the stretching vibration of C-H in the spectrum (Wang et al. 2016). The abundance of hydroxyl groups on the surface of MCQDs made the adsorbent well-hydrophilic. The MCQDs exhibited a slight shift in the characteristic Fe-O vibration peak compared with bare Fe_3_O_4_, indicating interaction between Fe_3_O_4_ and CQDs, which was consistent with literature reports (Mashkani et al. 2018; El Ghacham et al. 2025).

Thermogravimetry allowed further investigation of the thermal properties and composition of the nanocomposites (Fig. 1g). The thermal evolution of the MCQDs included three stages. A weight loss occurred at the first stage, attributed to evaporation of hydrogen-bonded water. Subsequently, mass loss occurred at the second stage. Additionally, weight loss was observed at the last stage, indicating combustion of the carbon skeleton in the CQDs. TG analyses indicated the successful binding of CQDs to Fe_3_O_4_.

Meanwhile, the pore structures of MCQDs were studied to investigate specific surface areas by BET analysis (Fig. 1h). The pore size of MCQDs was predominantly 3–5 nm, with a majority of micropores and mesopores. The adsorption isotherms of MCQDs all belonged to type IV isotherms, suggesting the occurrence of capillary condensation within the mesopores. The hysteresis loops observed were of the H2 type, which indicated a complex pore structure. Furthermore, the internal structure and surface characteristics of MCQDs revealed specific surface area was 98.25 m^2^/g, and the average pore size was 4.70 nm, which could facilitate fast mass diffusion.

### Magnetic recovery process

**Fig. 2:**
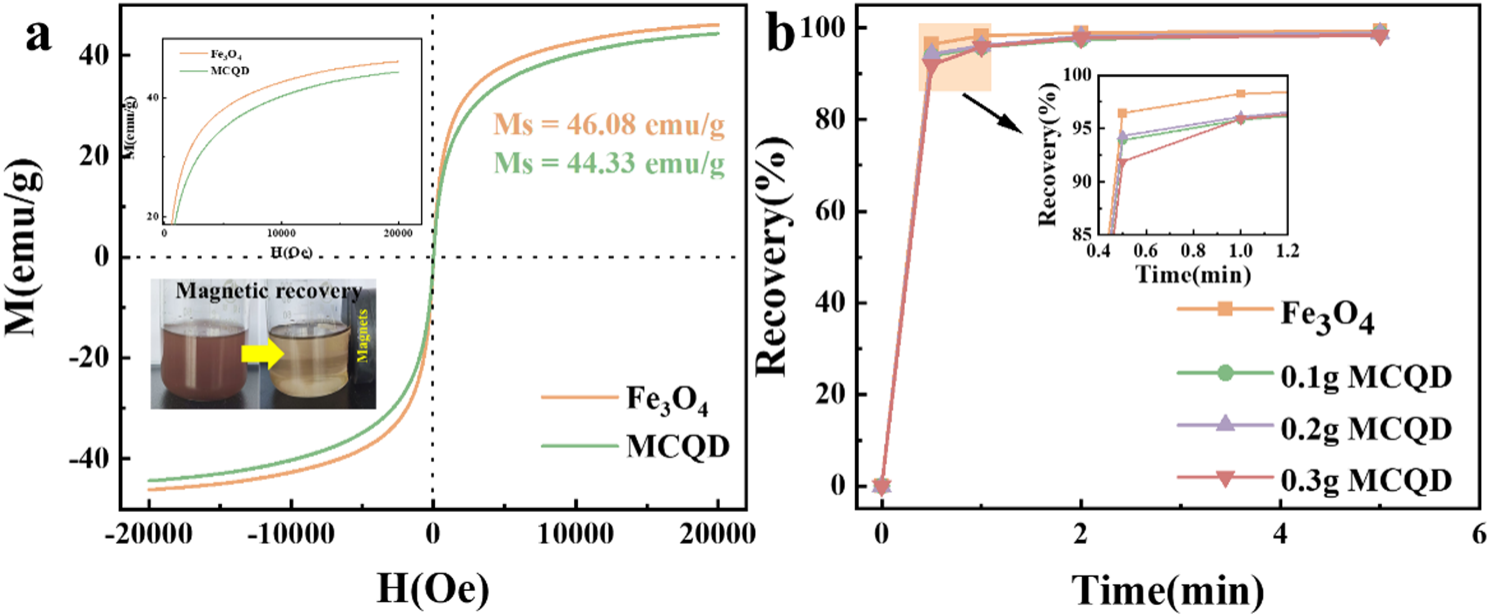
Magnetic hysteresis loops of MCQDs and bare Fe_3_O_4_ (**a**). The magnetic recovery kinetics of MCQDs and bare Fe_3_O_4_ (**b**)

Figure 2a showed that the magnetic hysteresis loops of MCQDs and Fe_3_O_4_ were both S-shaped curves, illustrating the superparamagnetism of MCQDs (Chen et al. 2012). The saturation magnetization (Ms) of MCQDs was 44.33 emu/g, which was less than Fe_3_O_4_ nanoparticle, which theoretically ensured that MCQDs can be demagnetized and dispersed in solution quickly after removal of the magnetic field. Magnetic recovery experiments showed that the aggregation of particles was quite slow by gravity alone, however, magnetic particles gathered rapidly in one direction upon application of a magnetic field. Once the external field was removed, the dispersion was quickly re-established by stirring slightly. Figure 2b showed the recovery efficiency of MCQDs with different contents in the presence of an external magnetic field. The trend of the recovery of suspensions with different concentrations was consistent, and the recovery efficiency was close to 100% after 5 min, illustrating the complete separation of MCQDs in solution via magnetic force in a short time, and the recovery rate was almost independent of the content of MCQDs.

### Adsorption kinetics

**Fig. 3:**
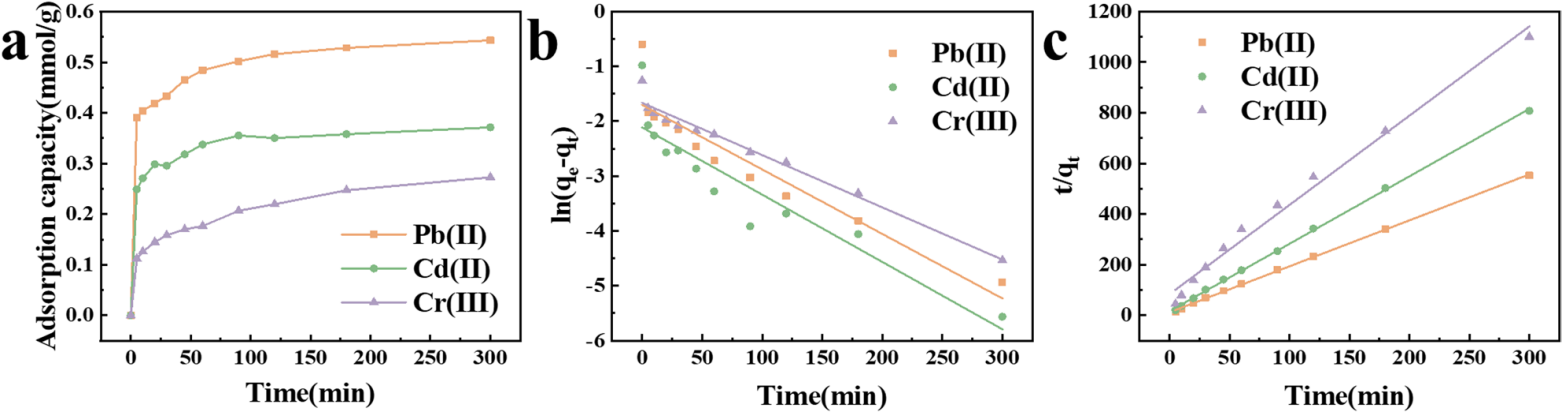
Adsorption kinetics of heavy metals on MCQDs (**a**), and fitting lines of pseudo-first-order model (**b**) and pseudo-second-order model (**c**)

The tendency of heavy metal adsorption capacity of MCQDs with time was shown in Fig. 3a. The adsorption kinetic curves of different metal ions were the same, and the metals adsorption equilibrium was reached after 3 h. For further understanding of the adsorption process and optimization of the adsorption conditions, the fitting of the heavy metal adsorption process to MCQDs was determined by the pseudo-first-order model (Eq. 3) and the pseudo-second-order model (Eq. 4), respectively (Bilgili 2006; Cheung et al. 2007).

3 $$ \ln \left( {q_{e} - q_{t} } \right)~ = ~\ln q_{e} - k_{1} t $$

4 $$ \frac{{\mathrm{t}}}{{{\mathrm{q}}_{{\mathrm{t}}} }}{\text{ = }}\frac{{\mathrm{1}}}{{{\mathrm{k}}_{{\mathrm{2}}} {\mathrm{q}}_{{\mathrm{e}}} ^{{\mathrm{2}}} }}{\text{ + }}\frac{{\mathrm{t}}}{{{\mathrm{q}}_{{\mathrm{e}}} }} $$

where *q*_e_ (mmol/g) was the adsorption capacity at final time; *q*_t_ (mmol/g) was capacity at specific time; *k*_1_ (min^−1^) and *k*_2_ (g/(mmol⋅min)) were the rate constant of models, respectively.

The kinetic fitting lines and the fitted kinetic data were presented in Fig. 3b–c; Table 1. The correlation of the pseudo-second-order equation was significantly better than the other one, which indicated the adsorption process can be explained by the pseudo-second-order kinetic model, which explains that the adsorption process was controlled by chemisorption.

**Table 1:** The parameters of the fitted kinetic data

		Pb (II)	Cd (II)	Cr (III)
Pseudo-first-order kinetic model	*q*_e_ (mmol/g)	0.1820	0.1210	0.1893
	k_1_ (min^−1^)	0.0118	0.0123	0.0095
	R^2^	0.8647	0.8375	0.9689
Pseudo-second-order kinetic model	*q*_e_ (mmol/g)	0.5505	0.3753	0.2836
	k_2_ (g/(mmol min))	0.2809	0.4459	0.1495
	R^2^	0.9993	0.9993	0.9861

### Adsorption isotherms

**Fig. 4:**
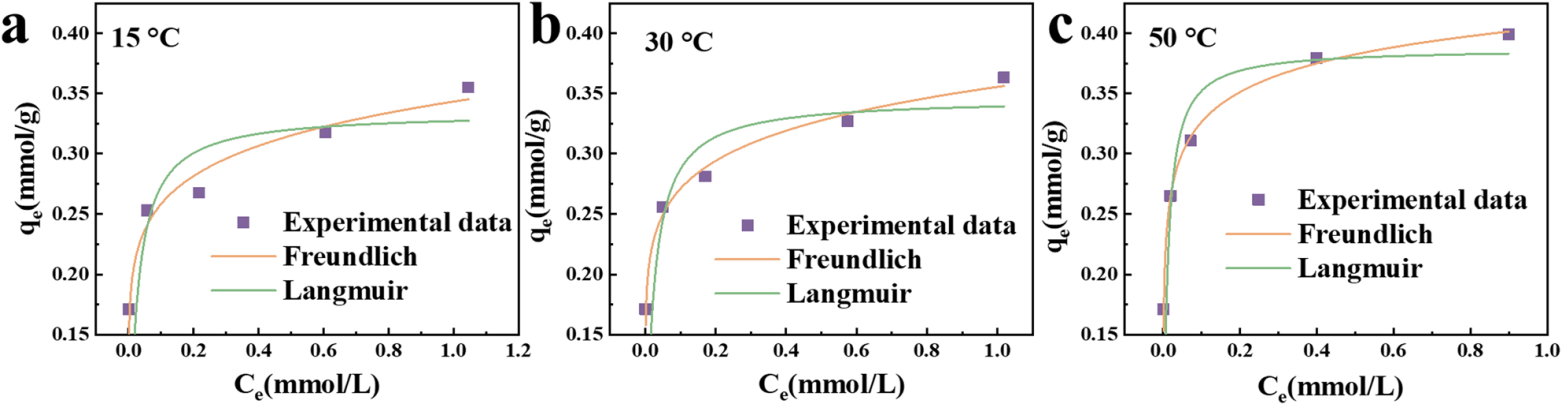
Adsorption isotherms for Cd (II) onto MCQDs at 15 °C (**a**), 30 °C (**b**), and 50 °C (**c**) fitted with Langmuir and Freundlich models

By varying the concentration of heavy metal ions, the relationships between the equilibrium adsorption capacity of Cd (II) by MCQDs and the equilibrium concentration at three temperatures were measured and were shown in Fig. 4a–c. The Freundlich equation exhibited a slightly higher degree of fit than the Langmuir equation. The parameters and correlation coefficients were listed in Table 2. Furthermore, the standard thermodynamic analysis for the adsorption of Cd (II) on MCQDs were calculated by Eqa. (5)–(7) (Lima et al. 2019). As shown in the results in Table 2, the ∆*G*^0^ value was smaller than zero, indicating that the spontaneous adsorption process of MCQDs can be proved by higher temperature. The positive ∆*H*^0^ values indicated the endothermic nature of the process, while the positive ∆*S*^0^ value suggested an increase in disorder at the solid-solution interface.

5 $$ \Delta G^{0} = - nRT $$

6 $$ \Delta G^{0} = - RT\ln K $$

7 $$ \ln K = \frac{{\Delta S^{0} }}{R} - \frac{{\Delta H^{0} }}{{RT}} $$

where *K* was the adsorption equilibrium constant, *n* was the dimensionless exponent of the Freundlich model.

**Table 2:** Adsorption isotherm and thermodynamics parameters for adsorption

		15 °C	30 °C	50 °C
Langmuir	*q*_m_ (mmol/g)	0.33	0.35	0.39
	K_L_ (L/mmol)	44.2009	48.7086	100.3626
	R^2^	0.4982	0.6634	0.8347
Freundlich	K_F_ [mmol/g (mmol/L)^−1/n^]	0.3433	0.3556	0.4049
	1/n	0.1236	0.1180	0.0770
	R^2^	0.8772	0.9595	0.9902
Thermodynamic parameters	∆G^0^ (kJ/mol)	-19.37	-21.35	-34.88
	∆H^0^ (kJ/mol)		110.55	
	∆S^0^ (J/mol K)		445.54	

### Heavy metal removal

**Fig. 5:**
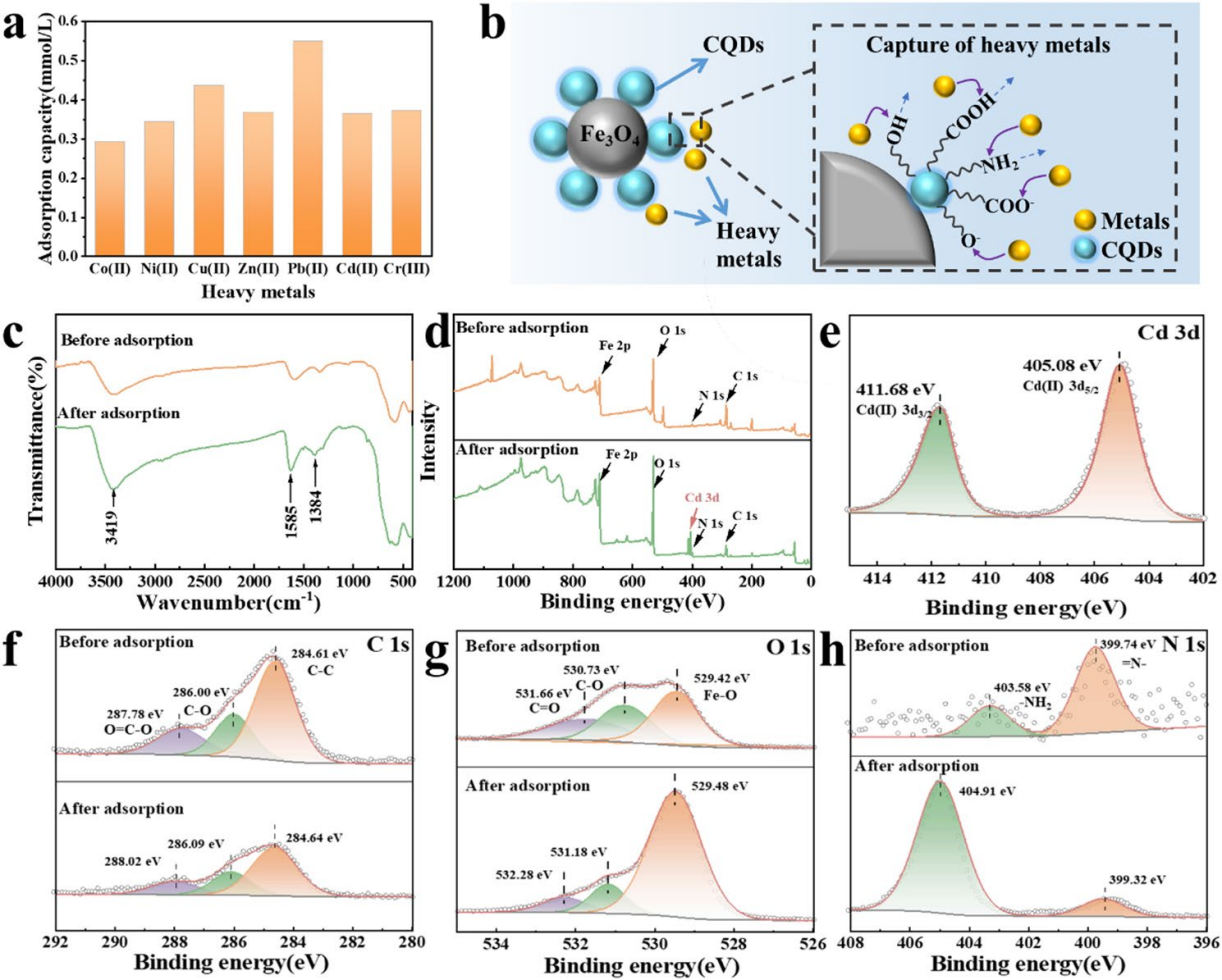
The adsorption performance for different heavy metals (a). The schematic of adsorption mechanism of MCQDs (b). The FT-IR spectra (c) and XPS full spectra (d) of MCQDs before and after Cd (II) adsorption. The XPS spectra of Cd 3d(e), C 1s (f), O 1s (g), and N 1s (h) of MCQDs before and after Cd (II) adsorption.

The adsorption equilibrium performance for different heavy metals was presented in Fig. 5a, demonstrating that the MCQDs have the substantial adsorption capacity for various heavy metal ions. Compared with previously reported magnetic carbon quantum dot adsorbents, MCQDs demonstrated superior or comparable adsorption performance (Table 3). MCQDs demonstrated excellent adsorption capabilities for heavy metals, and for elucidating the adsorption mechanism of MCQDs (Fig. 5b), FT-IR and XPS analyses were utilized. Figure 5c illustrated the FT-IR spectra of the sample before and after the adsorption of Cd (II). The peak of MCQDs after Cd (II) adsorption shifted to 3419 cm^−1^, indicating that the N-H or O-H groups may form interactions with the metal ions (Huang et al. 2019). Besides, for the MCQDs treated with Cd (II) ions, the peaks shifted to 1631 and 1384 cm^−1^, which was attributed to the links between Cd (II) and the carboxyl group present on MCQDs.

As shown in Fig. 5d, a new peak in the MCQDs was presented, which attributed to Cd (II) from the Cd 3d spectra (Fig. 5e) (Li et al. 2017). For C 1s spectra (Fig. 5f), the C-O and C=O peaks were shifted towards higher binding energy after adsorption, indicating C-O–Cd and C=O-Cd species were present. In addition, the O 1s spectra of samples (Fig. 5g) were composed of three types of peaks, including C=O (513.66 eV), C-OH or C-O-C (530.73 eV), and Fe-O (529.42 eV) (Huang et al. 2019). After the adsorption, the slight increment of binding energy could be attributed to the donation of electron density from oxygen atoms to cadmium ions (Luo et al. 2021). Meanwhile, the peaks attributed to -NH_2_ (404.91 eV) were obviously increased after Cd (II) adsorption in the N 1s spectrum (Fig. 5h). Based on the above findings, the adsorption mechanism of MCQDs was critically dependent on the interactive relationship between metal ions and oxygen-containing groups and amino groups.

**Table 3:** Summary of adsorption capacities of different magnetic CQDs materials

Adsorbent	Heavy metals	Adsorption capacity (mmol/g)	References
Fe_3_O_4_-PPCQDs	Cd (II)	0.1594	Pourshojaei et al. 2021
	Pb (II)	0.1146	
GSCQD-FeFe_2_O_4_	Pb (II)	0.1824	Saah et al. 2024
Magnetic CQDs	Zn (II)	0.4267	Ali et al. 2025
MCQDs	Co (II)	0.2923	This work
	Ni (II)	0.3438	
	Cu (II)	0.4356	
	Zn (II)	0.3676	
	Pb (III)	0.5488	
	Cd (II)	0.3634	
	Cr (III)	0.3726	

## Conclusion

In this study, MCQDs were successfully prepared for removing heavy metals by stable combination of CQDs derived from long-root *Eichhornia crassipes* with magnetic substances. Endowed with abundant oxygen-containing and amino groups functional groups on their surface, which provide ample active sites for heavy metal adsorption, MCQDs exhibited excellent adsorption capacity toward various heavy metal ions in aqueous solutions. Furthermore, the material demonstrated satisfactory magnetic recoverability, enhancing practical application in water treatment. These findings not only provided an efficient solution for heavy metal pollution treatment but also successfully promoted the value-added transformation and utilization of waste aquatic biological resources.

## Supplementary Information

Below is the link to the electronic supplementary material.

Supplementary Material 1

## Data Availability

The datasets used and/or analysed during the current study are available from the corresponding author on reasonable request.
