# Supplementary material for: Long-root Eichhornia crassipes waste plants dual-purpose resource utilization: green preparation of magnetic carbon quantum dots for heavy metal deep removal
Source: Bioresour Bioprocess. 2026 Apr 28;13(1):60. doi: 10.1186/s40643-026-01064-x (PMC13125687; doi:10.1186/s40643-026-01064-x)
Supplement: Supplementary file 1 — Supplementary Material 1 [file 40643_2026_1064_MOESM1_ESM.docx]

**Long-root *Eichhornia Crassipes* waste plants dual-purpose resource utilization: green preparation of magnetic carbon quantum dots for heavy metal deep removal**

Yihong Guo^1, 2^, Mingxin Cui^1*^, Hongjun Yang^3^, Jun Chen^2*^, Sen Lin^1, 2*^

*^1^Salt Lake Chemical Engineering Research Complex, Qinghai University, China*

*^2^National Engineering Research Center for Integrated Utilization of Salt Lake Resources, East China University of Science and Technology, China*

*^3^Yunnan Research Institute of Ecological Agriculture, China*

**Corresponding authors.*

*Email:* [*2013990005@qhu.edu.cn*](mailto:2013990005@qhu.edu.cn)*; chenjun2024@ecust.edu.cn; linsen@ecust.edu.cn***Supplementary information**


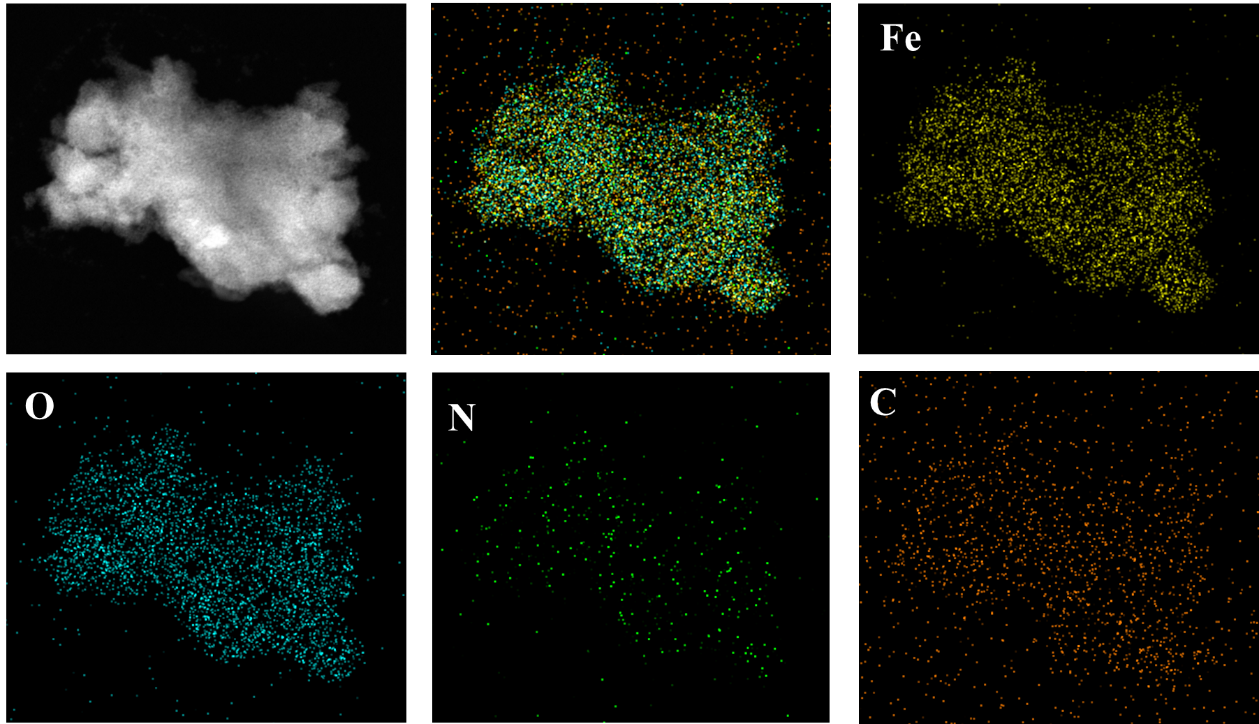


**Figure S1 The TEM image and elemental mapping of Fe, O, N, and C of MCQDs.**

**
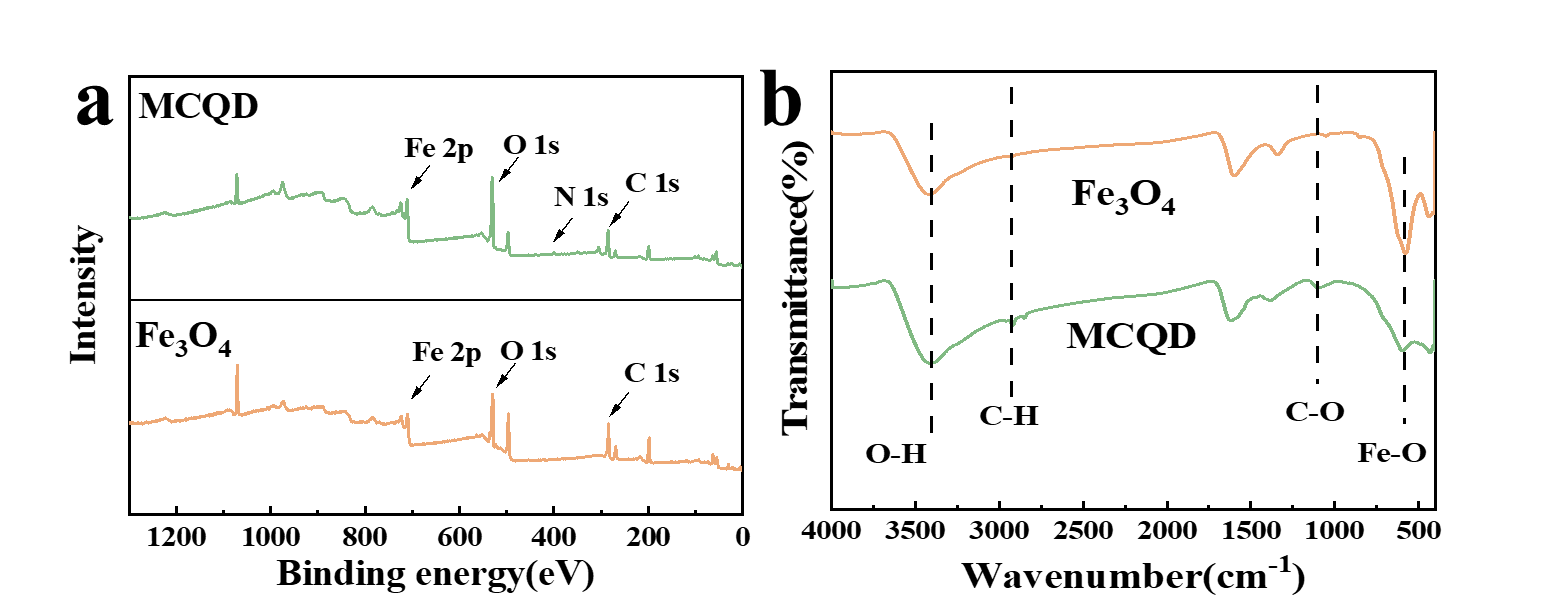
**

**Figure S2 The XPS full spectra (a) and the FT-IR spectra (b) of MCQDs and Fe_3_O_4_.**
